# Items

**Published:** 1882-09

**Authors:** 


### Items.

---

METEOROLOGICAL DATA.—For the month of July, 1882. H. Hall, Observer, U. S. Signal Station, Atlanta, Ga.

| | | |
|---|---|---|
| BAROMETER | —Mean | 30.076 |
| | Highest | 30.222 on 16th. |
| | Lowest | 29.774 on 4th. |
| | Range | 0.448 |
| THERMOMETER | —Mean | 75.2 |
| | Highest | 90.0 on 2d. |
| | Lowest | 57.8 on 6th. |
| | Range | 32.2 |
| | Greatest Daily Range | 21.7 on 15th. |
| | Least Daily Range | 8.7 on 22d. |
| Humidity | —Mean | 73.0 |
| Dew-point | —Mean | 65.1 |
| Rainfall | —Total | 6.6 inches. |
| | Greatest Daily | 1.80 on 3d. |
| | Total Number of Days | 16 |
| Wind | —Prevailing Direction | N. W. |
| | Maximum Velocity | W. 35 miles. |

WE depart from an established custom to invite attention to the announcement of Drs. Taliaferro and Noble, to be found among our advertising pages. We do this the more willingly because we believe we thereby do our readers a real service. The infirmary referred to was established by Dr. V. H. Taliaferro in the spring of 1881, and has been in successful operation since that time. The building occupied by the patients adjoins the residence of the physicians in charge, and the entire management is under their immediate supervision. A competent matron directs the affairs of the household, and an ample corps of efficient and experienced nurses—all white—are constantly employed.

When we consider the importance of close attention and the necessity for intelligent nursing in a large class of surgical and other uterine diseases, we feel confident that the institution now brought to the notice of the medical profession will be fully appreciated and liberally patronized.

---

THE *Medical Chronicle* is the name of a new monthly journal, edited by our valued friend, George H. Rohe, M.D., of Baltimore, the first number of which is before us. We gladly extend to the *Chronicle* a most cordial greeting, and tender our wishes for a prosperous career.

The first number gives promi.e of a vigorous constitution, and we doubt not, under Dr. Rohe's management, that the enterprise will be made a conspicuous success.

We regret, however, to note an error, by implication rather than by direct assertion, in regard to the early history of ether anæsthesia which occurs in an interesting article on "Anæsthetics and their administration," by the editor.

It was not the Boston chemist, as he asserts, but a modest, unobtrusive Georgia gentleman who first demonstrated the anæsthetic properties of ether, and to him should be given the credit of this inestimable boon which he has conferred upon humanity.

---

A CONTRIBUTOR to the *Buffalo Medical and Surgical Jour-*

*nal* condemns indiscriminate forced spinal extension preliminary to fixation in cases of spinal curvature. He contends that injury is liable to result, and indeed testifies that it has followed the improper application of extension. Referring to the plaster of Paris jacket he says: "The cause for such misapplication of what, properly used, is a valuable adjunct to the mechanical treatment of spinal disease, is due to two facts: 1st. Indefinite ideas on the part of many practitioners of the nature and effect of vertebral ostitis, and an inability to recognize the different stages of the disease; 2d. To the imperfect manner in which the method that includes suspension, has been promulgated."

---

THE town authorities of Brighton, England's famous health resort. are suing the *London Lancet* for alleged libellous publications concerning the sanitary, or rather unsanitary condition of the place. Thirty thousand dollars, it is said, has been guaranteed to prosecute the suit. That amount judiciously expended would do much, doubtless, toward removing the causes which served as a basis for the strictures on the salubrity of the town.

---

PARIS has a commission for regulating the height of buildings, which are graded to correspond to the width of the street upon which they front. Houses may be forty feet high on streets twenty-five feet wide. In no case are they permitted to be over sixty-five feet high, and only then when the streets are sixty-five feet wide or wider.

---

IT has become necessary in New York to pass a stringent law prohibiting the establishment and maintenance of "opium dens."

---

ABOUT this time look out for marvelous successes in the treatment of consumption by the use of paraciticides.

---

DR. T. GAILLARD THOMAS is again in his old chair at the College of Physicians and Surgeons, New York.

